# The synergistic effect of acupuncture and computer-based cognitive training on post-stroke cognitive dysfunction: a study protocol for a randomized controlled trial of 2 × 2 factorial design

**DOI:** 10.1186/1472-6882-14-290

**Published:** 2014-08-07

**Authors:** Shanli Yang, Haicheng Ye, Jia Huang, Jing Tao, Cai Jiang, Zhicheng Lin, Guohua Zheng, Lidian Chen

**Affiliations:** Rehabilitation Hospital Affiliated to Fujian University of TCM, Fuzhou, 350003 China; Rehabilitation Medicine College, Fujian University of Traditional Chinese Medicine, Fuzhou, 350122 China; Academy of Integrative Medicine, Fujian University of Traditional Chinese Medicine, Fuzhou, 350122 China; Fujian University of Traditional Chinese Medicine, Fuzhou, 350122 China

**Keywords:** Acupuncture, Computer-based cognitive training, Stroke, Cognitive dysfunction

## Abstract

**Background:**

Stroke is one of the most common causes of cognitive impairment. Up to 75% of stroke survivors may be considered to have cognitive impairment, which severely limit individual autonomy for successful reintegration into family, work and social life. The clinical efficacy of acupuncture with Baihui (DU20) and Shenting (DU24) in stroke and post-stroke cognitive impairment has been previously demonstrated. Computer-assisted cognitive training is part of conventional cognitive rehabilitation and has also shown to be effective in improvement of cognitive function of affected patients. However, the cognitive impairment after stroke is so complexity that one single treatment cannot resolve effectively. Besides, the effects of acupuncture and RehaCom cognitive training have not been systematically compared, nor has the possibility of a synergistic effect of combination of the two therapeutic modalities been evaluated. Our primary aim of this trial is to evaluate the synergistic effect of acupuncture and RehaCom cognitive training on cognitive dysfunction after stroke.

**Method/Design:**

A randomized controlled trial of 2 × 2 factorial design will be conducted in the Rehabilitation Hospital Affiliated to Fujian University of Traditional Chinese Medicine. A total of 240 patients with cognitive dysfunction after stroke who meet the eligibility criteria will be recruited and randomized into RehaCom training group, acupuncture group, a combination of both or control group in a 1:1:1:1 ratio. All patients will receive conventional treatment. The interventions will last for 12 weeks (30 min per day, Monday to Friday every week). Evaluations will be conducted by blinded assessors at baseline and again at 4, 8 and 12 weeks. Outcome measurements include mini–mental state examination (MMSE), Montreal cognitive assessments (MoCA), functional independence measure scale (FIM) and adverse events.

**Discussion:**

The results of this trial are expected to clarify the synergistic effect of acupuncture and RehaCom cognitive training on cognitive dysfunction after stroke. Furthermore, to confirm whether combined or alone of acupuncture and RehaCom cognitive training, is more effective than conventional treatment in the management of post-stroke cognitive dysfunction.

**Trial registration:**

Chinese Clinical Trial Registry: ChiCTR-TRC-13003704.

Registration date: 4 September, 2013.

## Background

Stroke is the leading cause of death worldwide [1]. Perhaps more importantly, those who survive stroke have various neurologic deficits, including physical and motor skill impairment and serious psychological consequences. The latter may affect cognition, impairing processes such as attention and concentration, memory, planning, calculations and language as well as emotions and behaviors [2, 3]. Cognitive impairment is one of the most common disorders caused by stroke [4–7]. Approximately 25% of patients present with cognitive impairment 3 months after a stroke. Furthermore, up to 75% of stroke survivors may be considered to have cognitive impairment [8–10]. After stroke, the risk of developing dementia doubles, and the progression rate from mild cognitive impairment to dementia increases [11, 12]. Cognitive impairment after stroke is, therefore, an unfortunately common problem and will likely become more of one as people live longer [13].

Despite a high prevalence of post-stroke cognitive impairment, therapeutic possibilities are still limited. Several life style interventions [such as smoking cessation, moderation of alcohol intake, healthy diet (e.g., Mediterranean diet), weight control and physical activity] have been used in clinical practice,which potentially can help to prevent progression or occurrence of vascular cognitive impairment. Nonetheless, there are still no FDA-approved treatments for cognitive impairment after stroke [14]. By and large, drug trials have failed to show improvement in global cognitive functioning, activities of daily living, and quality of life [14]. Treatment options for cognitive impairment after stroke remain limited and poorly supported [13].

As a result, complementary or alternative therapies, such as acupuncture, are attractive to both patients and practitioners. Acupuncture, a core component of Traditional Chinese Medicine (TCM), has been used for thousands of years in several oriental countries to treat various diseases, with virtually no side effects in competent hands. A number of studies [15–21] demonstrated that acupuncture techniques may be effective for improving intelligence, stimulating consciousness and enhancing memory. These effects may result from activation and/or stimulation by acupuncture of the regions responsible for cognition. In the system of TCM, Baihui (DU 20) and Shenting (DU24) are acupoints that belong to the Du Meridian and may be important in the nervous system. Acupoint Shenting (DU24) is considered to be involved in the improvement of human health and spirits, and Baihui (DU20) in the adjustment of memory function. Therefore, the Baihui and Shenting acupoints are commonly used in China to clinically treat post-stroke cognitive impairment [22, 23]. A series of experimental studies have demonstrated that acupuncture can improve cognitive function through: 1. regulating the aging related changes in gene profile expression of the hippocampus [24]; 2. preventing oxidative stress [25]; 3.regulating the expression of apoptosis related genes in hippocampus [26, 27].

Cognitive rehabilitation (CR) is a mechanism that can compensate for an impaired nervous system by systematic treatment that can cause functional changes through the reinforcement, promotion and relearning of previously learned or new patterns to enhance cognitive function [28]. At present, cognitive training mainly includes traditional cognitive retraining and computer-assisted cognitive training, e.g. [29]. Computer-assisted cognitive training allows automation of many cognitive training procedures, making it possible to meet demand for care, improve stimulation quality, increase patient record reliability and optimize performance in impaired functions [30, 31].

RehaCom is one of the most widely used computer-assisted cognitive training software packages, enabling focus on various cognitive areas that need to be trained [32], which has shown excellent results in clinical practice with no major negative effects reported [33]. To date, the RehaCom system has been widely used in relevant diseases rehabilitation [34, 35]. The RehaCom system promises a series of advantages over traditional cognitive training, on the basis of pen-and-paper exercises. The RehaCom program has sufficient flexibility, simplicity, accessibility, dynamics and objectivity to make a useful contribution to clinical practice. Its interactive capabilities and multimedia make it possible to keep patients motivated, irrespective of multiple motor and sensory deficits. Its use enables more precise recording of patient results and increased quality of stimulation. It also allows setting the initial level of task difficulty according to the individual’s baseline competency and gradually increasing it as patients improve their performance, resulting in a continuous cognitive challenge. In addition, RehaCom enables the standardization of intervention [33].

As mentioned above, the acupuncture focus on the global cognitive function from the holistic conception of TCM, while the RehaCom cognitive training concerns the specific impaired cognitive functions which need to be trained from the viewpoint of symptomatic treatment. However, the cognitive impairment after stroke is so complexity that one single treatment cannot resolve effectively. Due to the limited efficacy of the interventions separately used and the growing interest in recovery from post-stroke cognitive dysfunction has led to development of multiple therapeutic strategies for cognitive rehabilitation, that is, the remediation or alleviation of cognitive deficits resulting from neurological damage [36]. As the two interventions which can treat the cognitive impairment from global cognitive function to specific impaired cognitive function, we firmly believe that the interaction of multiple therapeutic strategies (the combination of acupuncture and RehaCom cognitive training) to produce an effect greater than simply the sum of the individual effects of each intervention if they were used separately. Besides, the effects of acupuncture and RehaCom cognitive training have not been systematically compared, nor has the possibility of a synergistic effect of combination of the two therapeutic modalities been evaluated.

Nonetheless, after reviewing a great amount of literature, we found that few studies had addressed these questions. So it is still unclear whether this form of intervention (acupuncture combined with RehaCom cognitive training) can be considered as a treatment of choice. Though, a current systematic review [37] showed that acupuncture in combination with other therapies could significantly improve cognitive functions. Besides, acupuncture would be a useful tool in conjunction with RehaCom from our preliminary clinical practice. However, convincing evidence is still inadequate. Therefore, we designed a randomized controlled trial of 2 × 2 factorial design to address these problems and hopefully provide a more conclusive answer to the questions. We hypothesized that the synergistic effect of acupuncture and RehaCom cognitive training is greater than simply the sum of the individual effects of each intervention if they were used separately; and the acupuncture or RehaCom cognitive training significantly improves cognitive outcomes, or to a similar extent, assessed after the intervention, compared to the control group.

In this trial, our first aim is to evaluate the synergistic effect of acupuncture and RehaCom cognitive training on cognitive dysfunction after stroke. Our second aim is to determine whether acupuncture and RehaCom cognitive training, alone or in combination, are more effective than conventional treatment on the rehabilitation of post-stroke cognitive dysfunction. The work reported in this article is financed by the Program of International S&T Cooperation (No. 2011DFG33240), Ministry of Science and Technology of the People’s Republic of China.

## Method/Design

### Study design and setting

**Figure 1:**
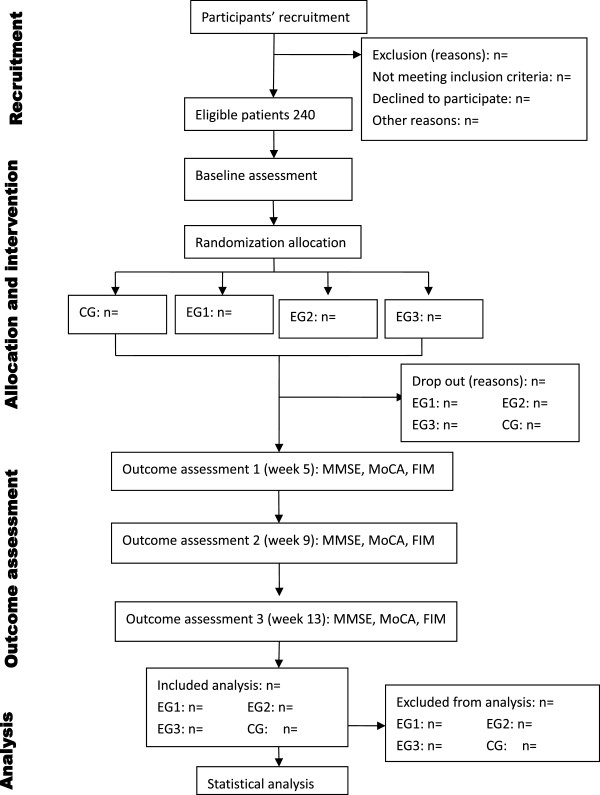
**Flow diagram of participants.**

We propose a randomized controlled trial with 2 × 2 factorial design in which the two interventions (acupuncture and RehaCom training) are to be delivered to **four** groups defined by the presence or absence of each intervention, with one arm without both interventions as a control group. Patients were randomized to one of the four treatment arms: (i) Experimental Group1 (EG1): conventional treatment plus RehaCom cognitive training; (ii) Experimental Group 2 (EG2): conventional treatment plus acupuncture treatment; (iii) Experimental Group 3 (EG3): conventional treatment plus the combination of acupuncture and RehaCom cognitive training; and (iv) Control Group (CG): Conventional treatment alone. All groups receive the same intervention time of 30 minutes. The investigation will have an intervention period of 12 weeks and will be carried out in the Rehabilitation Hospital Affiliated to Fujian University of Traditional Chinese Medicine (FJTCM). Cognitive abilities screening instrument on cognition performance [includes mini-mental state examination (MMSE), Montreal cognitive assessment (MoCA), functional independence measure scale (FIM)] will be assessed in all groups before and after randomization (at 0,4,8 and 12 weeks). The flow diagram for this trial is presented in Figure 1.

### Sample size

We use the improvement of MMSE scores as the main effect indicators to estimate the sample size. Sample size calculations are performed to determine the number of participants needed to detect effect sizes. Based on the similar papers reported before [38–40] and our preliminary data indicates the mean with SD of the MMSE scores were μ1 = 2.7, μ2 = 5.9, μ3 = 3.7, μ4 = 6 in the CG, EG1, EG2, EG3 respectively, with the SD *σ* = 5.2. To detect 4.6 mean differences among the groups at end of interventions, k represents of the number of groups which is 4 in this trial. About 50 individuals per group will be calculated according to the formula:


With a type I error of 5% (α = 0.05) and 90% power (β = 0.10). Estimating a 20% dropout rate during the study, a minimum of 240 total participants is needed to reach the target 60 participants per group.

### Participant and recruitment

We will recruit 240 eligible patients from the Rehabilitation Hospital Affiliated to FJTCM who suffer from cognitive disorders caused by stroke with age above 18 years. The eligible patients must meet the following inclusion criteria as well as the exclusion criteria. A CONSORT diagram of participant recruitment is shown in Table 1. Patients will be recruited as inpatients or from the outpatient therapy department of the rehabilitation hospital by their treating therapist, through an advertisement at the hospital’s homepage, and through flyers at several locations of the hospital. Interested patients can contact the project leader (PL) through their therapist, by phone, email, or mail. If an applicant meets the study criteria, he or she will be invited to the research.

**Table 1:** **Trial processes chart**

Items	Before enrollment	Intervention period 1	Outcome assessment 1	Intervention period 2	Outcome assessment 2	Intervention period 3	Outcome assessment 3
	-2-1(week)	1-4(week)	5(week)	5-8(week)	9(week)	9-12(week)	13(week)
Inclusion criteria	×						
Exclusion criteria	×						
Informed consent	×						
Baseline	×						
Randomization and allocation	×						
Intervention		×		×		×	
Mini-mental status examination	×		×		×		×
Montreal cognitive assessment	×		×		×		×
Functional independence measure scale	×		×		×		×
Adverse events		×		×		×	
Reasons of drop-out and withdrawals		×		×		×	

### Inclusion criteria

For inclusion, patients (age between 18 and 75 years old) within six months after first-ever stroke (according to the criteria of ‘Diagnostic Criteria for All Kinds of Cerebrovascular Disease’ [41]) with cognitive impairments [screened by Mini-Mental Status Examination (MMSE) [42]: illiterate (uneducated) group ≤ 17points; primary school (years of education ≤ 6 years) group ≤ 20 points; middle school or higher (years of education > 6 years) group ≤ 24 points]. Conscious, stable physical condition and signed informed consent of patient.

### Exclusion criteria

Criteria for exclusion are as following: pre-existing psychological disorders will be excluded; severe visual and/or hearing problems (impede the operation of RehaCom cognitive training system); Inability to follow instructions(impede cognitive training or assessment); Pregnant and lactating women (it is still unclear whether acupuncture will lead to abortion or other side effects); Bleeding disorder (the acupuncture procedure involves penetration of the skin, therefore we will exclude participants with thrombocytopenia, and so on); Serious illnesses of the heart, liver, or kidney, or other severe diseases (there may be risks that acupuncture induces stress action and thus harms patients with serious illness); Participating in other clinical trials that would affect the evaluation results of this study.

### Randomization and allocation

The random allocation sequence will be produced by an independent statistician via the PLAN sentences of the statistical software SAS9.1, who works in the Evidence-Based Center of the university and is not involved in the study. The eligible patients will be randomly and blindly allocated to the study groups (EG1, EG2, EG3, CG) to ensure internal validity of results according to 1:1:1:1(equal proportion rule). The random allocation sequence will be managed by a specified research assistant (RA) who is not involved in this trial and be concealed to the screeners and assessors until the last outcome assessment of the patient has been performed. The treating therapists (acupuncturist) will be notified of the allocation and arrange directly with the patient for the allocated treatment to be given. The eligible participants will be informed which group they are in by the project manager.

### Blinding

Blinding of study personnel in research projects is a main quality criterion of a study [43]. Nevertheless, blinding of therapists in an intervention study who perform experimental treatment in patients is not always feasible, especially if experimental intervention shall be combined with in the therapy. Consequently, hiding the patient’s allocation in this setting will not be possible. Therefore, neither therapists nor patients will be blinded in this study. We will assign a specified RA to be in charge of the management of the random allocation sequence and blind code of allocation in which the intervention group (CG, EG1, EG2, EG3) will be replaced by the alphabet A, B, C or D. In this trial, we will assign each investigators a well-defined obligation: the research assistant and treating therapists (acupuncturist) will be not involve in the assessment of outcomes; The outcome assessors and the statistic analyzer will be not involved in the participants’ screening and allocating. The blind code will be disclosed after the statistical analysis is completed.

### Treatment allocation

The intervention period in this trial will conduct at a frequency of 30 min per day (Monday to Friday every week), 12 weeks in total. Precise documentation of therapy/treatments, medication and the related lifestyle influence factors will be recorded in the case report forms (CRFs). The groups and intervention allocation listed in Table 2.

**Table 2:** **Groups and intervention allocation**

Groups	Interventions
Control group(CG)	Conventional treatment
RehaCom training(EG1)	Conventional treatment + RehaCom cognitive training
Acupuncture group(EG2)	Conventional treatment + acupuncture treatment
Combination group(EG3)	Conventional treatment + acupuncture treatment + RehaCom training

### Conventional treatment

#### Basic treatment

The participants in the all groups will receive basic treatments which are usually delivered in Rehabilitation hospitals Affiliated to FJTCM. The doctor in charged will decide what the treatment or should be according to the ‘*Chinese prevention and treatment guidelines of cerebrovascular disease*’ [44]. This means that these patients will receive the treatment that the doctor considers to be the most necessary treatment includes conventional treatment of internal medication and multidisciplinary treatment which includes physiotherapy, occupational therapy, medical exercise therapy, hydrotherapy etc., except cognitive training. There are no restrictions for this condition and we will track the types of interventions provided and precise documentation of therapy treatments, medication, and therapy time/day.

#### Health education

The doctor in charged will base on the blueprint of ‘*Out of The Misunderstanding of the Rehabilitation of Stroke Patients*’ [45] (the first prize of Popular science books from the Chinese Association of TCM)to health education the patients to change their lifestyle and the concept on the rehabilitation of stroke. Two strategies will be used for health education: (1) Educational brochures on stroke, published by FJTCM, will be sent to participants. Information in these brochures will include the concept, epidemiology, etiology, common symptoms, diagnosis, treatments and precautions that should be taken. (2) The rehabilitation therapist will introduce and explain the contents of the educational brochures to all participants in person. The aim is to help participants learn about and understand stroke and to implement strategies to help alleviate the disease. The frequency (quantity) of related factors applied to the patients will be precisely documented in the CRFs.

### RehaCom cognitive training

The computer-based cognitive training program, Hasomed RehaCom (CE certified, EN-ISO-13485) is intended for patients with acquired cognitive deficits after brain damage, e.g. after a stroke [46]. The RehaCom [33] software has five different therapeutic groups aimed at restoration of attention, memory, executive functions, and visual field, respectively. The developer (International Cooperator-Reha Rheinfelden) has helped to provide a Chinese revised version of the software. The program contains several modules with different levels of difficulty, automatically increasing task difficulty level as the subject successfully executes simpler procedures. Recording of number of errors and test completion time for all patients and a training results file enable continuity over several sessions and database storage of results. The computer gives patients appropriate instructions and feedback on performance in their own language (in our patients Chinese). The following were the areas of training: divided attention, concentration, reaction (time), word memory, verbal memory (i.e., whole text, not just individual words), spatial memory and figure memory. Application of the training sessions can be learnt after a short instruction. This will also be part of the training workshop prior to commencement of the study. Patients’ training used RehaCom software for interactive computerized cognitive rehabilitation, a complete description of which has been published [47].

### Acupuncture protocol

For every treatment visit, participants are treated with 2 acupuncture points [Baihui (DU20)] and [Shenting (DU24)]. Patients will be treated by an experienced acupuncturist with a working experience of at least five years in neurological rehabilitation with acupuncture. The detail of acupoints showed in Table 3.

**Table 3:** **Acupuncture points selected in this protocol**

Acupuncture points	Location
Baihui (DU20)	On the head, 5 *B-cun superior to the anterior hairline, on the anterior median line.
	Note 1: DU20 is located in the depression 1 *B-cun anterior to the midpoint of the line from the anterior hairline to the posterior hairline.
	Note 2: When the ears are folded, DU20 is located at the midpoint of the connecting line between the auricular apices.
Shenting (DU24)	On the head, 0.5 *B-cun superior to the anterior hairline, on the anterior median line.

*B-cun: Proportional bone cun. This method divides the height of the human body into 75 equal units. Using joints on the surface of the body as the primary landmarks, the length and width of every body part is measured by such proportions.

#### Definition of acupuncture

Acupuncture originated in ancient China approximately 2,500 years ago and is a key component of TCM. Acupuncture techniques involve insertion of needles and the stimulation of the needles may involve manual, electrical, heat, laser or other forms of stimulation [48].

#### Needling method

Points are first sterilized with alcohol, and then the doctor’ s left hand must fix the target position, the right hand insert the thin, disposable acupunctures needle (Huatuo, single use, φ0.35 × 40 mm, distributor Suzhou HuaTuo Medical Instruments, Suzhou, China) at angle of insertion 10° ~ 20°(between needle and scalp) to a depth of approximately 0.3-0.5B-cun. Following insertion, stimulation of the acupuncture point will be performed using of bidirectional rotation of the needle sleeve, to achieve the sensation known as *Deqi*, which is commonly described as a ‘glowing’ feeling. The needle will be maintained in place for 30 minutes, with bidirectional rotation of the needle sleeve (with amplitude and rotation speed as stipulated previously) for one minute, every 10 minutes (a total of four such rotations per session). Following the treatment session, the needles will be withdrawn.

### Acupuncture combined RehaCom cognitive training

For the combined group, patients will receive acupuncture needling and training with the RehaCom cognitive system while the needles inserted (at the same time).

### Outcome assessment

This study will run for 12 weeks and cognitive function will be assessed by neuropsychological measurement scales which include Mini–mental state examination (MMSE), Montreal Cognitive Assessment (MoCA), Functional Independence Measure Scale (FIM) at baseline and again at 4, 8 and 12 weeks (within a time window: +/- 3 days). All outcomes will be measured by several experienced assessors who are blinded to the randomization group after the baseline visit in the Evaluation Department of Rehabilitation Hospital Affiliated to FJTCM.

### Primary outcomes

#### Mini–mental state examination (MMSE)

The mini–mental state examination (MMSE) or Folstein test [49] is a brief 30-point questionnaire test that is used to screen for cognitive impairment. MMSE includes items to evaluate spatial and temporal orientation, short-term memory, attention and calculation abilities, language, thinking, and action planning. MMSE will take about 15 to 20 minutes. In this study we use the Chinese version of MMSE [42] to follow the course of cognitive changes in an individual over time, thus making it an effective way to document an individual’s response to treatment.

### Secondary outcomes

#### Montreal cognitive assessment (MoCA)

The MoCA [50] test is a one-page 30-point test administered in approximately 10 minutes assesses several cognitive domains: visual-executive, naming, attention, language, abstraction, delayed recall, and orientation. It makes up the shortcoming of MMSE to have a comprehensive evaluation on the cognitive changes in the patients with different degrees of cognitive impairment. We use the Beijing version 26 August, 2006 translated by Wei Wang & Hengge Xie [51].

#### Functional independence measure scale (FIM)

The FIM [52] is a detailed measurement of functionality tool used to evaluate the functional status of patients throughout the rehabilitation process following a stroke [53]. The FIM’s assessment (We use the Chinese version translated by Yulong Wang [42]) of degree of disability depends on the patient’s score in 18 categories, focusing on motor and cognitive function which may reflect global cognitive function [54]. So, FIM scores may be interpreted to indicate level of independence or level of burden of care.

### Patient safety

Adverse effects attributable to the RehaCom therapy may include mental fatigue or headache. Symptoms usually resolve in patients as they progress with therapy and become more familiar with it [33]. Acupuncture may cause discomfort or bruising at the sites of needle insertion, nausea, or feeling faint after each treatment.

During the trial, all unexpected adverse events (AEs) will be observed in detail and documented in CRFs. All AEs reported should be analyzed regardless of the investigators’ assessments of causality. If serious adverse events happen, the researchers should report to the PL and ethics committee immediately, who will make a decision on whether the participant needs to withdraw from the study.

### Data collection and management

Having sought consent, the demographic and baseline characteristic data in CRFs will be completed by a specific RA who is masked to treatment allocation and trained to administer questionnaires in a standardized way.

The data consists [55] of demographic details, review of medical history and medication, the lesion, affected side, handedness, cognitive function, time since stroke, stroke type and subtype, rehabilitation history (treatments until study start), details of current and cognitive treatment received within the previous 3 months and concomitant medications are recorded. Data will be obtained by direct questions to patients, from medical records, and from a relative or proxy.

Outcome measurement will be measured by the outcome assessors after the intervention. RAs will conduct quality control of data collection and be responsible for data entry. The PL will be responsible for initial data cleaning, identifying, coding, and conversion into the proper format for data analysis.

All forms must be dated and signed by the responsible investigator or one of his/her authorized staff members.

### Statistical analysis

All allocated subjects will be analyzed with the available data, i.e. on the basis of the intention-to-treat (ITT). A significance level of 95% (two sided p-values alpha < 0.05) will be used. After examining the data for normality (Kolmogorov-Smirnov test) the outcome measures will be analyzed using ANOVA (assuming normal distribution) or Kruskal-Wallis (non-parametric). Demographic characteristics and other baseline values will be described using descriptive statistics for each group. Significant group interactions will be analyzed post-hoc. We will use the Bonferroni method to appropriately adjust the overall level of significance for multiple outcomes.

### Quality control

- A trained RA will explain to each participant the nature of the study, its purpose, the procedures involved, the expected duration, the potential risks and benefits and any discomfort it may entail. Patients or their legal guardians will receive an information letter about the study and there will be no time limit for them to consider implications, ask questions, and respond to the invitation to participate. Participants will be informed that they are free to withdraw from the treatment under study at any stage for any reason without any consequences.
- The participants screening must according to the inclusion and exclusion criteria. If they meet the inclusion criteria and not meet the exclusion criteria, this procedure will pass. On the contrary, the disqualified participants will be excluded, and the researchers will record the number and the reasons.
- The random allocation sequence will be produced by an independent statistician via the statistical software SAS 9.1. The project manager allocates the participants according to the random allocation sequence, and it must be deposited for safe keeping ensuring nobody else knows. The cessation of blinding will take place only after all the trial is completed.
- The missing data should not appear in the CRFs of the missing data in principle. If any missing data appears, researchers should find out whether it is missed in the raw data. If it is missed when loading into database, it should be reloaded. If the missing data affects outcome measure, the participant will be rejected.
- To ensure that treatments are of a high standard and delivered in accordance with the trial protocol, clinicians involved in assessments, treatment or training will have to provide proven record of at least three year of clinical experience and certified training or education in related fields of rehabilitation or research. The needling will only be performed by professional physicians with specific training in TCM and a minimum of five years of experience. They will participate in a two-day training in the standard operating procedures (SOPs) provided by the author of the manualized protocol and the standard operation videos. In this training the protocol will be explained and practiced on each other during exercises and role-plays. The therapist in the control condition will receive no extra training. In addition, all treatments provided to patients will be carefully recorded in the CRFs.
- Blinding of study personnel in research projects is a main quality criterion of a study [43]. Although patients, investigators, and therapists administering treatment will not be blinded, those assessing the patients for primary and secondary endpoints will be blinded to patient treatment assignment. The investigators, therapists and assessors are different people. Patients will be told not to talk to the examiner about the group allocation or therapy content during the post-intervention assessments.
- To maintain the quality of this trial, monitoring will be conducted by the PL located in the Rehabilitation Hospital Affiliated to FJTCM. Investigators can also be convened to discuss practical issues that might be encountered, such as dealing with serious adverse events, revising the protocol, and addressing certain important issues that might be raised by investigators and participants.

### Ethics issue

This study is conducted in accordance with the *Declaration of Helsinki*. All participants will be fully informed about the trial, and will sign the written informed consent form prior to participation. The trial is approved by the Ethics Board of the Rehabilitation Hospital Affiliated to FJTCM (2013KY-004-02).

## Discussion

The study is a single-blind, randomized controlled trial of 2 × 2 factorial design mainly to evaluate the synergistic effect of acupuncture and RehaCom cognitive training in patients with cognitive disorders caused by stroke. The trial is sponsored and financially supported by the Program of International Science and Technology Cooperation (No. 2011DFG33240), which is the most important projects to promote national S&T innovation capabilities based on international cooperation.

Cognitive impairment is a condition characterized by mental deficits. Up to 75% of stroke survivors may be considered to have cognitive impairment [8]. These disorders severely limit individual autonomy for successful reintegration into family, work and social life [4]. Despite a high prevalence of post-stroke cognitive impairment, therapeutic possibilities are still limited. So far, complementary or alternative therapies seem to have some positive effect in the post-stroke cognitive impairment (e.g. acupuncture [17, 40] and computer based cognitive training [56]). But, improvements in cognitive function tend to be modest. Besides, currently there is not enough evidence for acupuncture [57] or computer-assisted cognitive training [56] based on strict clinical trial of evidence-based medicine (EBM) because of the poor quality of current studies, for example, small sample size, no description of methods for randomization, no standardized acupuncture protocol, no strict quality control which may lead to performance bias, and so on.

Besides, the cognitive impairment after stroke is so complexity that one single treatment cannot resolve effectively. Additionally, the effects of acupuncture and RehaCom cognitive training have not been systematically compared, nor has the possibility of a synergistic effect of combination of the two therapeutic modalities been evaluated. Due to the acupuncture focus on the global cognitive function from the holistic conception of TCM, while the RehaCom cognitive training concerns the specific impaired cognitive functions which need to be trained from the viewpoint of symptomatic treatment. So, we believe that the interaction of multiple therapeutic strategies (the combination of acupuncture and RehaCom cognitive training) to produce an effect greater than simply the sum of the individual effects of each intervention if they were used separately. Nonetheless, the convincing evidence is still lacking. Therefore, our trial aims to clarify these issues and hopefully provide a more conclusive answer to the questions by the means of randomized factorial controlled trial.

We used cognitive abilities screening scales includes MMSE, MoCA and FIM as the outcome measures in order to track changes in the functional status over the 12-week interventions period. The MMSE [49] is a global measure of cognitive function that has been used extensively both as a diagnostic tool for dementia screening as well as a cognitive outcome measure for gauging the efficacy of the cognitive alterations, for example [58, 59].The MoCA was designed as a rapid screening instrument for mild cognitive dysfunction, which has been found to have high sensitivity and specify for the detection of mild cognitive impairments in various neurological disorders (e.g. Stroke [60]). The FIM was designed to assess disability based on the International Classification of Function (ICF). It is a useful measure of physical and cognitive disabilities in patients with AD and VaD which may reflect global cognitive function [54]. Therefore, they can be a comprehensive neuropsychological test battery to monitor the general cognitive alterations which can reach a more reliable conclusion.

The biggest limitation of this protocol is that it is not double-blinded. However, the outcome assessors and statistical analysts will be blind to the intervention to decrease possible bias to ensure the quality of this trial. The study also lacks long-term follow-up and evaluation. The 12-week intervention period reflects what is done in actual clinical practice and is long enough to test whether a rehabilitation modality is effective in the short term and whether the efficacy is sustained during the follow-up period.

In conclusion, the results of this trial are expected to clarify the synergistic effect of acupuncture and RehaCom cognitive training on cognitive dysfunction after stroke. Furthermore, to confirm whether combined or alone of acupuncture and RehaCom cognitive training, are more effective than conventional treatment in the management of post-stroke cognitive dysfunction.

### Trial status

Ongoing recruitment.
